# Supplementary material for: Electrospun Scaffolds of Polylactic Acid, Collagen, and Amorphous Calcium Phosphate for Bone Repair
Source: Pharmaceutics. 2023 Oct 25;15(11):2529. doi: 10.3390/pharmaceutics15112529 (PMC10674189; doi:10.3390/pharmaceutics15112529)
Supplement: Supplementary file 1 [file pharmaceutics-15-02529-s001.zip › FIGURE-S1-eps-converted-to.pdf]

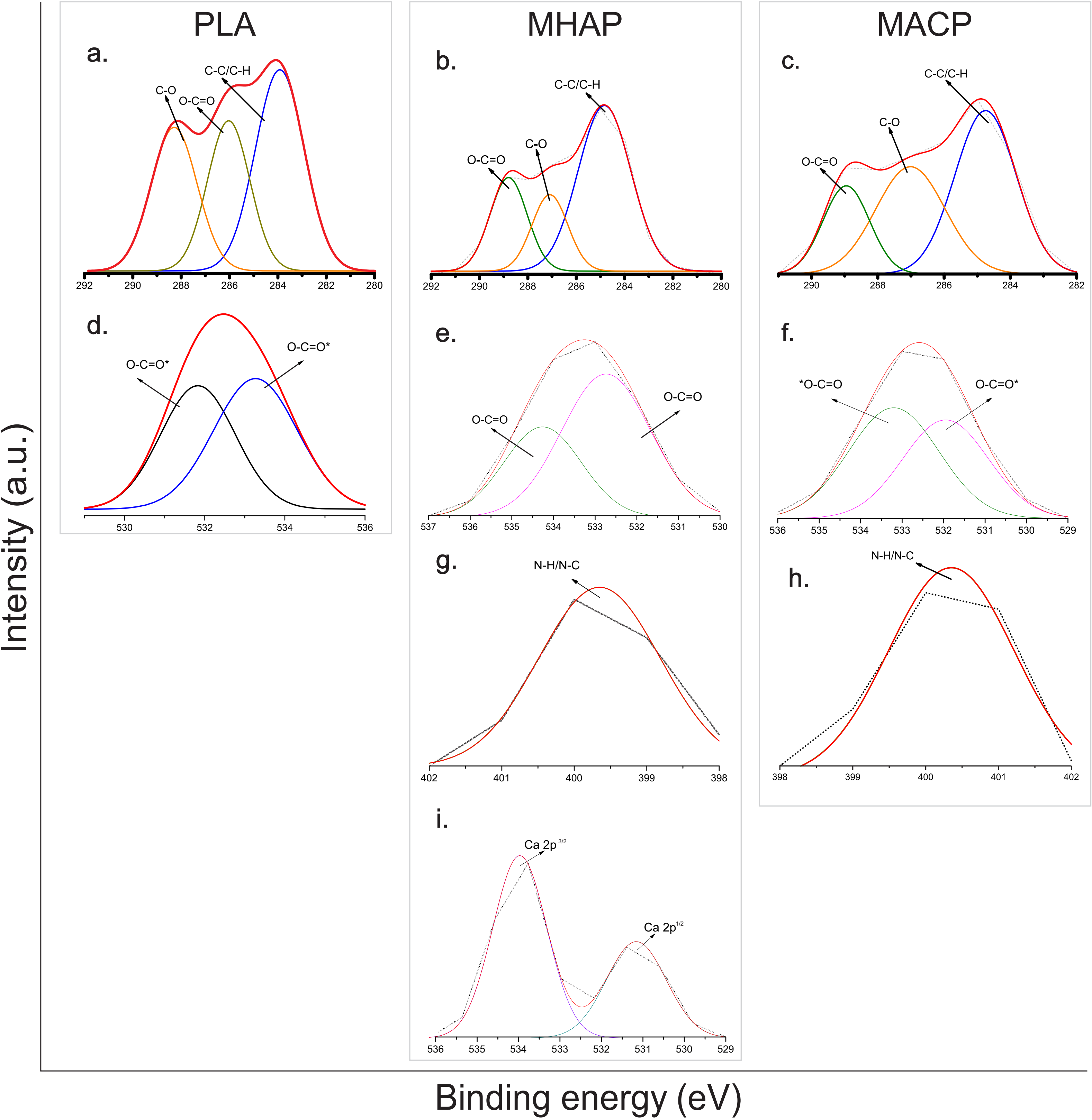

Figure S1: Deconvolution of the C1s, O1s, N1s and Ca2p signals of PLA (a and d), MHAP (b, e, g and i) and MACP (c, f and h) electrospun scaffolds.
