# Supplementary material for: Electrospun Scaffolds of Polylactic Acid, Collagen, and Amorphous Calcium Phosphate for Bone Repair
Source: Pharmaceutics. 2023 Oct 25;15(11):2529. doi: 10.3390/pharmaceutics15112529 (PMC10674189; doi:10.3390/pharmaceutics15112529)
Supplement: Supplementary file 1 [file pharmaceutics-15-02529-s001.zip › FIGURE-S2-eps-converted-to.pdf]

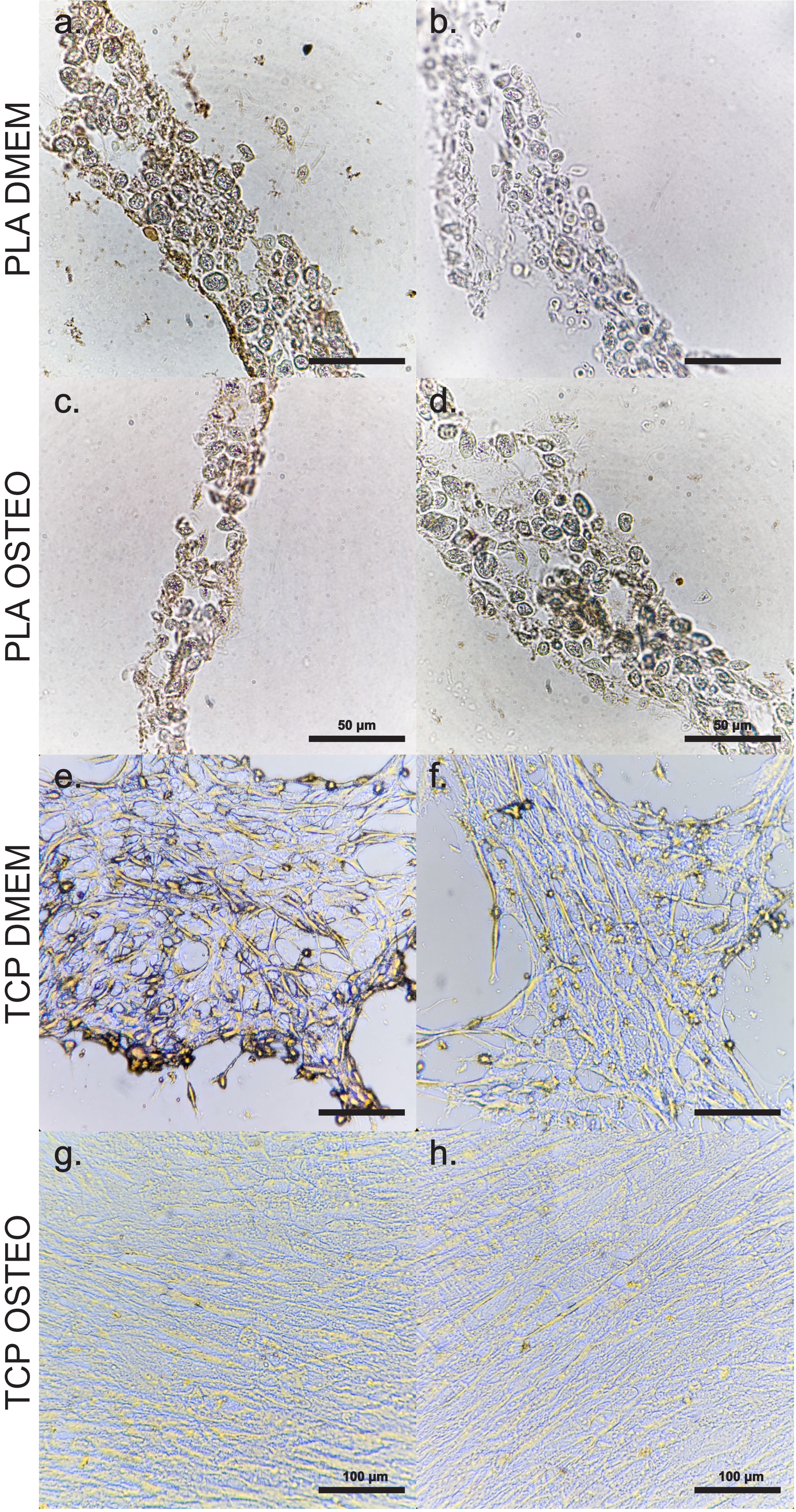

Figure S2. OCN immunohistochemical staining of hWJ-MSCs cultured on PLA scaffolds. Images (a) and (c) show positive staining for OCN in cells exposed to supplemented DMEM culture medium, while images (b) and (d) show negative staining. Images (e) and (g) show positive staining for OCN in cells in TCP exposed to osteogenic differentiation medium, while images (f) and (h) show negative staining.
